# The Impact of Long-Term Air Pollution Exposure on Type 1 Diabetes Mellitus-Related Mortality among U.S. Medicare Beneficiaries

**DOI:** 10.3390/toxics11040336

**Published:** 2023-03-31

**Authors:** Trenton J. Honda, Fatemeh Kazemiparkouhi, Helen Suh

**Affiliations:** 1 School of Clinical and Rehabilitation Sciences, Northeastern University, Boston, MA 02115, USA; 2 Department of Civil and Environmental Engineering, Tufts University, Medford, MA 02155, USA

**Keywords:** type 1 diabetes, air pollution, chronic exposure, particulate matter, nitrogen dioxide

## Abstract

Background: Little of the previous literature has investigated associations between air pollution exposure and type 1 diabetes mellitus (T1DM)-related mortality, despite a well-established link between air pollution exposure and other autoimmune diseases. Methods: In a cohort of 53 million Medicare beneficiaries living across the conterminous United States, we used Cox proportional hazard models to assess the association of long-term PM_2.5_ and NO_2_ exposures on T1DM-related mortality from 2000 to 2008. Models included strata for age, sex, race, and ZIP code and controlled for neighborhood socioeconomic status (SES); we additionally investigated associations in two-pollutant models, and whether associations were modified by participant demographics. Results: A 10 μg/m^3^ increase in 12-month average PM_2.5_ (HR: 1.183; 95% CI: 1.037–1.349) and a 10 ppb increase in NO_2_ (HR: 1.248; 95% CI: 1.089–1.431) was associated with an increased risk of T1DM-related mortality in age-, sex-, race-, ZIP code-, and SES-adjusted models. Associations for both pollutants were consistently stronger among Black (PM_2.5_: HR:1.877, 95% CI: 1.386–2.542; NO_2_: HR: 1.586, 95% CI: 1.258–2.001) and female (PM_2.5_: HR:1.297, 95% CI: 1.101–1.529; NO_2_: HR: 1.390, 95% CI: 1.187–1.627) beneficiaries. Conclusions: Long-term NO_2_ and, to a lesser extent, PM_2.5_ exposure is associated with statistically significant elevations in T1DM-related mortality risk.

## 1. Introduction

Type 1 diabetes mellitus (T1D), which makes up approximately 5% of all diabetes mellitus, is an autoimmune disease that is differentiated from type 2 diabetes mellitus (T2D) by a unique pathophysiology, risk factors, and comorbidity profile. While T2D is a disorder characterized by insulin resistance, T1D is characterized by the autoimmune destruction of pancreatic beta cells caused by an environmental trigger in genetically susceptible individuals, resulting in profound insulin deficiency and hyperglycemia [1]. Importantly, T1D is associated with dramatic premature mortality from renal and cardiovascular complications between 20 and 30 years after disease onset, with one study finding a standardized mortality ratio of 3.6 (95% CI 3.3–3.9) relative to individuals without T1D [2]. As the incidence and prevalence of T1D is increasing globally, by an estimated 5% per year in the US, it is imperative that modifiable risk factors which may exacerbate the morbidity and mortality associated with this condition are identified [1].

While air pollution has been increasingly and consistently linked to T2D incidence, prevalence, and perturbations in disease control and progression [2,3,4], associations between air pollution and T1D are much less studied. Almost all of the extant literature to date explores the impact of air pollution on pediatric T1D incidence, prevalence, and glycemic control, with mixed results [5,6,7]. As T1D is a chronic and progressive disease associated with high excess mortality from long-term complications, the dearth of studies examining the impact of air pollution on late-stage disease outcomes, such as T1D mortality, significantly limits the understanding of the public health impact of air pollution on T1D. 

Prior studies provide mechanisms which may explain how air pollutants impact T1D mortality risk. Air pollution exposure has been previously associated with increased glycosylated hemoglobin levels among T1D patients [8]. Poor glycemic control in T1D has also been identified as a major determinant of excess mortality, with a recent study in Sweden finding that even small elevations in HbA1c among T1D patients were associated with excess cardiovascular (CV) mortality, with the highest glycosylated hemoglobin (HbA1c) levels (>9.7%) having 10.46 times the risk of CV death (95% CI 7.62–14.37) relative to non-T1D patients [9]. 

To address these gaps in the literature, we examined the associations between ambient PM_2.5_ and NO_2_ exposure and T1D-related mortality in a national cohort of US Medicare beneficiaries.

## 2. Materials and Methods

### 2.1. Air Pollution Exposure 

#### 2.1.1. PM_2.5_

We estimated 12-month moving average PM_2.5_ concentrations using well-validated GIS-based (Geographical Information System) spatiotemporal models that estimated daily PM_2.5_ exposure on a 6 km grid covering the conterminous US [10]. Model inputs included PM_2.5_ data from the US Environmental Protection Agency (EPA), meteorological and geospatial covariates, and traffic-related PM estimated using a Gaussian line source dispersion model. The daily PM_2.5_ model performed well, with high precision, as evidenced by a cross-validation R^2^ of 0.76, a low mean of the absolute value of the prediction errors (CVMAE), and low bias, as shown by a normalized mean bias factor (NMBF) close to zero and a slope of measured against the model predictions close to one.

#### 2.1.2. NO_2_

We estimated 12-month moving average NO_2_ concentrations using land use regression models developed by Bechle, Millet [11], that estimated monthly NO_2_ exposure for census blocks using land use regression (LUR) and spatially varying temporal scaling factors to estimate monthly NO_2_ concentrations on a 100 m grid across the conterminous US from 2000 to 2008. Estimates had high validity and low error, explaining 81% of the spatial (R^2^ = 0.81), 73% of temporal (R^2^ = 0.73), and 84% of the spatiotemporal variation (R^2^ = 0.84) in monthly mean NO_2_ concentrations.

### 2.2. Population Data

We compiled enrollment data from the Centers for Medicare and Medicaid Services for 53 million Medicare beneficiaries (ages 65–120) living in the conterminous US between 2000 and 2008. For each enrollee, we obtained beneficiary-specific information on date of birth, sex (as a binary variable), race (as Asian, Black, Hispanic, White), ZIP code of residence, and survival. We categorized age into 1-year intervals, with 90+ years included as 1 age interval to avoid excessive zero counts. Using the International Classification of Disease (ICD-10) codes from the National Death Index, we extracted mortality from type 1 diabetes (E10).

### 2.3. Data Linkage

For PM_2.5_ and NO_2_, we matched beneficiaries to the grid point closest to the ZIP code centroid of each beneficiary’s reported residential address, accounting for residential moves. As our main exposure window of interest, we assessed the impact of 12-month moving average exposure for both pollutants of interest. While all participants had valid PM_2.5_ measures assigned to their ZIP code of residence, NO_2_ estimates were available only for 91.2% of the Medicare population. As such, for NO_2_, we employed complete-case analyses.

### 2.4. Covariates

Covariates were selected based upon their prior associations with diabetes mortality or air pollution. Individual level covariates included age, sex, and race/ethnicity. Area-level covariates included ZIP code and state-level SES, which were assessed using the annual mean gross adjusted income from the US Internal Revenue Service (IRS) Statistics of Income Division database [12]. Urbanicity (urban vs. nonurban) was assessed using Categorization B from the Rural Health Research Center (RHRC) [13]. For a subset of our Medicare population, we linked measures from Selected Metropolitan/Micropolitan Area Risk Trends of the BRFSS (Behavioral Risk Factor Surveillance System), which provide data on health-related risk behaviors for 378 US counties. In our analysis, 28.4 million beneficiaries lived in ZIP codes (13,893 of 38,715) located in a county with BRFSS data. Covariates available for this sub-population included monthly county-level prevalence of current smokers, non-White people, heavy drinkers (i.e., >two drinks per day), asthma, and mean body mass index.

### 2.5. Statistical Analysis

We examined the associations between 12-month moving average PM_2.5_ and NO_2_ exposure and diabetes-related mortality in single- and two-pollutant models using Cox proportional hazards (Cox PH) models, with strata for age, sex, race (White/non-White), and ZIP code, controlling for ZIP code and state SES. We examined effect modification using interaction terms for age, sex, race, urbanicity, and urban ZIP code SES categories. All results are expressed as the hazard ratio (HR) per 10 μg/m^3^ and 10 ppb increase in 12-month average PM_2.5_ and NO_2_, respectively. Monthly county-level covariates were included in models for all exposure windows. We implemented all Cox models in Java, incorporating linkage, grouping-and other data mining techniques to reduce memory needs and improve computational efficiency [14].

#### Sensitivity Analysis

In sensitivity analyses, in our BRFSS sub-population, we additionally assessed the association of PM_2.5_ and NO_2_ exposure and mortality, adjusting for ZIP code and state SES. This was performed both with and without adjusting for potential confounding by monthly county-level prevalence of current smokers, non-White race, heavy drinkers (i.e., >two drinks per day), asthma, and mean body mass index. Additionally, we examined whether PM_2.5_-associated HRs varied with the length of the exposure window, examining the association of PM_2.5_ exposure based on 24-, 36-, 48-, and 60-month moving averages.

## 3. Results

Our study population included approximately 53 million Medicare enrollees living in nearly 39,000 US ZIP codes between 2000 and 2008 (Table 1). During the study period, more than 32,000 T1DM deaths were reported. The overall mean 12-month PM_2.5_ concentration was 10.32 μg/m^3^ (sd = 3.15), and the mean 12-month NO_2_ concentration was 10.9 ppb (sd = 5.6).

### 3.1. Associations with PM_2.5_

**Figure 1:**
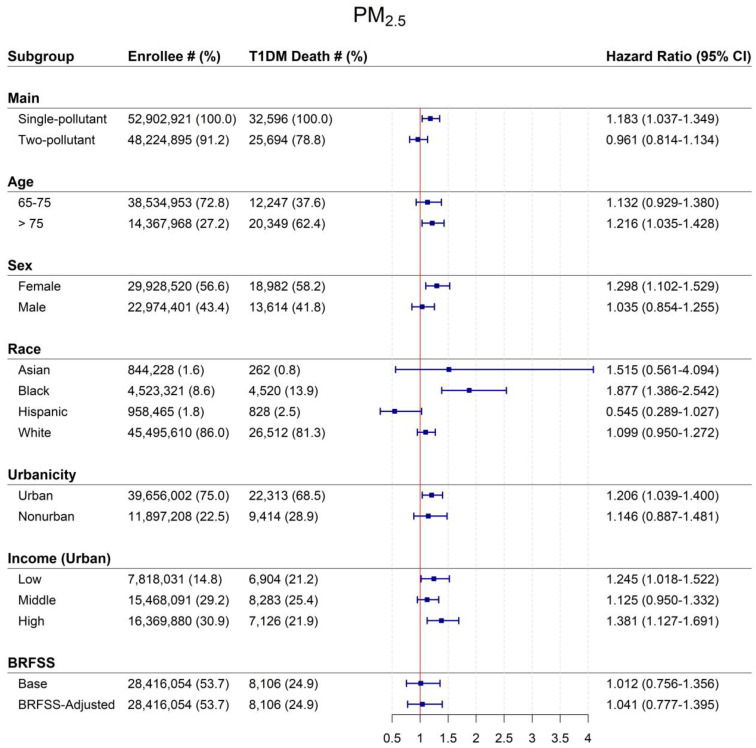
Mortality hazard ratios (95% CI) associated with a 10 μg/m^3^ increase in 12-month average PM_2.5_ for entire population and by subgroup, US 2000–2008. Abbreviations: CI = confidence interval; PM_2.5_ = particles with aerodynamic diameters < 2.5 μm; T1DM = type 1 diabetes mellitus. BRFSS data were not available for all of the Medicare participants included in our full analyses. As such, the sample size for these sub-analyses is smaller.

Figure 1 shows HRs associated with 12-month PM_2.5_ for T1DM mortality for the entire population and effect modification models for subgroups. In single-pollutant models, a 10 μg/m^3^ increase in exposure to PM_2.5_ increased the risk of dying from type 1 diabetes by 18.3% (HR:1.183, 95% CI: 1.037–1.349). PM_2.5_-associated HRs increased with longer moving averages (Table 2), with the highest HRs observed for 60-month PM_2.5_ moving average exposure (HR: 1.527, 95% CI:1.235–1.889). When models were additionally adjusted for NO_2_ in two-pollutant models, associations were no longer statistically significant (HR 0.961, 95% CI: 0.814, 1.134). 

We found risks of death to vary by beneficiary characteristics (Figure 1). Type 1 diabetes-related mortality risks differed most by race, with Black beneficiaries having the highest HRs [1.877 (95% CI: 1.386–2.543)]. While risks were elevated for Asian and White participants, they were not statistically significant. Women had higher HRs [1.298 (95% CI: 1.102–1.529)] compared to men [1.035 (95% CI: 0.854–1.255)]. By age, HRs were higher for participants >75 years [1.216, 95% CI 1.035–1.428) compared to younger (65–75) beneficiaries [1.132, 95% CI 0.929–1.380).

Neighborhood characteristics also demonstrated associations with PM_2.5_ and type 1 diabetes mortality. Beneficiaries living in urban compared to nonurban ZIP codes had higher mortality risks. We found generally similar PM_2.5_-associated risks for low- and high-income ZIP codes in urban areas. However, PM_2.5_-associated risks were null for beneficiaries living in middle-income ZIP codes.

### 3.2. Associations with NO_2_

**Figure 2:**
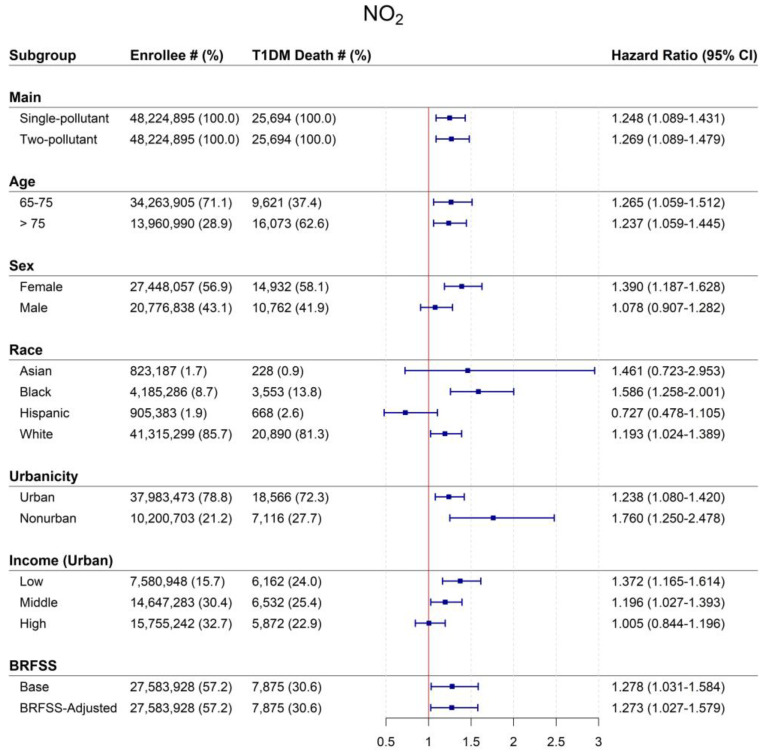
Mortality hazard ratios (95% CI) associated with a 10 ppb increase in 12-month average NO_2_ for entire population and by subgroup, US 2001–2008. Abbreviations: CI = confidence interval; NO_2_ = nitrogen dioxide; T1DM = type 1 diabetes mellitus. BRFSS data were not available for all the Medicare participants included in our full analyses. As such, the sample size for these sub-analyses is smaller.

Figure 2 shows significantly elevated HRs associated with 12-month NO_2_ for type 1 diabetes-related mortality for the entire population effect modification models for subgroups, with a 10 ppb increase in NO_2_ increasing risk of dying from type 1 diabetes by 24.8% (1.248, 95% CI: 1.089–1.431). In models controlling for PM_2.5_, the NO_2_-associated type 1 diabetes mortality was largely unchanged (HR: 1.269, 95% CI: 1.089–1.479].

As in our PM_2.5_ models, we observed NO_2_-associated risks of death to vary by race, with Black beneficiaries again having the highest HRs (1.586, 95% CI: 1.258–2.001). Unlike our PM_2.5_ models, White beneficiaries had positive and significant NO_2_-associated mortality risks (HR: 1.193, 95% CI: 1.024–1.389); associations for Asian participants were again positive but not statistically significant. Women had higher HRs (1.390, 95% CI: 1.187–1.628) compared to men (1.078, 95% CI: 0.907–1.282). By age, we found generally similar NO_2_-associated risks for both age groups.

Neighborhood characteristics modified the associations between NO_2_ and type 1 diabetes mortality as well. Beneficiaries living in nonurban compared to urban ZIP codes had nominally higher mortality risks. However, when limited to urban areas, we found higher risks of increased type 1 diabetes-related mortality for beneficiaries living in low (HR: 1.372, 95% CI: 1.165–1.614)- compared to high (HR: 1.005, 95% CI: 0.844–1.196)-SES ZIP codes. In sensitivity analyses adjusting for BRFSS variables, associations did not significantly nor importantly differ from the same population in unadjusted models.

## 4. Discussion

To our knowledge, we are the first to investigate the associations between air pollution and type 1 diabetes mortality in a national cohort of older Americans. We found large and significant associations between NO_2_ and, to a lesser extent, PM_2.5_ and type 1 diabetes mortality, which was robust in the adjustment for multiple important potential confounders and in the use of different exposure windows. For both pollutants, associations were significantly and importantly higher among Black and female participants, while for other variables (i.e., urbanicity and income), associations varied between pollutants.

While no studies to date have examined the impact of air pollutant exposures on T1DM mortality in adults, our findings are consistent with some, but not all, of the minimal prior literature exploring long-term air pollution’s impact on T1DM in children. In a recent study, Lanzinger et al. (2022) found long-term PM_2.5_ and PM_10_ exposure to be significantly associated with increased HbA1c levels in a cohort of child and adolescent T1DM patients in Germany [8]. Importantly, they also found that both pollutants were significant and important risk factors for severe hypoglycemic episodes [8], which is an acute T1DM complication that has previously been associated with upwards of 11% increased mortality risk [15]. While it is unclear whether hypoglycemia is a result of air pollution exposure or a symptom of other underlying illnesses, it has been postulated that the impact of air pollution exposure on glycemic control may lead T1DM patients to administer higher insulin doses, increasing their risk of severe hypoglycemia [16] and, by extension, mortality. These findings were not, however, consistent with other studies, which found no association between PM and NO_2_ and either insulin dose or HbA1c. [6,17] Importantly, all the prior literature has explored the impact of air pollution in children and adolescents, which is a very different population than the one we examined, and no prior studies have examined mortality as an outcome.

Our findings are largely consistent with the growing literature showing PM_2.5_ and NO_2_ to be associated with perturbed glucose metabolism among adult diabetic populations. In our prior work on undifferentiated diabetes mellitus in an older US population, we observed NO_2_ and PM_2.5_ exposure to have strong and consistent associations with elevated HbA1c levels (2.0% ± 0.7%, *p* < 0.01, and 1.8% ± 0.6%, *p* < 0.01, respectively) [3,4]. Others have observed similar findings of air-pollution-mediated perturbations in glucose metabolism in Chinese [4], Korean [18,19], and European populations [20]. However, most of these studies examined undifferentiated diabetes mellitus and presumed that their study populations comprised >95% T2DM, and thus may not be directly analogous to our study population. However, while the pathophysiology of T1DM differs dramatically from T2DM, the underlying mechanisms by which air pollution induces abnormal glucose metabolism and increased mortality risk may be similar. For example, pulmonary oxidative stress [21] and the resultant systemic inflammatory response [22] that has been observed in animal and human studies of air pollution could impact insulin resistance and serum glucose control, affect the amount of self-administered insulin, increase the risk of hypoglycemic episodes (which have recently been linked directly to CVD risk and mortality [23]), and/or directly increase the burden of CVD in T1DM patients [24]. Indeed, the most common cause of death among T1DM patients is CVD, a cause of death that has been causally linked to air pollution exposure [25,26].

We also found that associations between PM_2.5_ and NO_2_ and T1DM mortality differed by race and sex. Hazard ratios for PM_2.5_ and NO_2_ among Black participants were 70.8% and 32.9% higher than those for White participants, respectively. Importantly, this finding is consistent with the prior literature identifying disparities in T1DM glucose control and clinical outcomes by race, which underpins the broad impact of structural racism on health in racial and ethnic minority groups [27,28]. Consistent with this, the growing literature demonstrates that Black populations experience higher cause-specific mortality risk from air pollution exposure relative to other racial/ethnic groups [29,30,31]. These risk disparities underpin the broad and detrimental impacts of structural racism, and the resultant differential access to healthcare and differential exposure to environmental toxicants [32]. It is unclear why we found consistent differential mortality risk by sex, with females consistently having higher mortality risk for both pollutants. However, the prior literature on sex differences in air pollutant mortality risk have found that these differences are often outcome specific [33].

Our study has a number of important limitations that should be considered when interpreting our findings. In terms of exposure, while our pollutant estimates are all based upon well-validated models, it is likely that nondifferential exposure misclassification bias effects our estimates toward the null in the present study. Additionally, all participants in our study were adults  > 65 years living in the US. As such, it is likely that our findings are not universally generalizable to non-US populations or younger populations with T1DM. Third, while we had information on individual-level age, sex, and race/ethnicity, we were unable to adjust for potential individual level SES or behavioral confounders. Last, it is possible that there is outcome misclassification in the recording of T1D on death certificates. While prior studies have shown that type 2 diabetes recorded on death certificates has high specificity (98.1%) [34,35] and variable sensitivities (30–50%), no prior studies have examined type 1 diabetes mellitus recorded on death certificates. As the studies on type 2 diabetes mellitus have found higher rates of diabetes reporting when examining beyond the first listed cause of death on the death certificate [35], the sensitivity of our data extraction using the four leading causes of death for T1D will likely be higher. These prior studies on T2D also found higher rates of reporting as the duration of the underlying illness increased. As T1D is most commonly a disease that begins in childhood, the disease would be well represented in the medical records of our Medicare participants. Additionally, unlike T2D patients, T1D patients are often enrolled in regional or state registries [36], which likely increases the likelihood of accurate recording on death certificates. These limitations are counterbalanced by a number of important strengths. Our cohort’s large size and use of exposure models to estimate PM_2.5_ exposures for each ZIP code allowed for sufficient statistical power to estimate associations for T1D, an understudied and relatively rare disease, as well as examine understudied racial/ethnic populations in effect modification models.

## 5. Conclusions

In the large population of older US adults, we found long-term NO_2_ and, to a lesser extent, PM_2.5_ exposure to be associated with significant and important elevated risks of T1DM-related mortality. Associations with NO_2_ were of large magnitude, while both pollutants associations were strongest among Black and female participants. As T1D is increasing in incidence and prevalence across the globe for reasons that are unclear, it is important to identify modifiable predictors of mortality in this group. Our findings suggest that air pollution may be an as-yet-unidentified risk factor for mortality among individuals with T1DM.

## Figures and Tables

**Table 1:** Baseline demographics for Medicare beneficiaries and death time demographics for diabetes-related death.

	Beneficiaries	Death due to Type 1 Diabetes
Persons, *n* (%)	52,902,921 (100.0)	32,596 (100.0)
ZIP code, *n* (%)	38,715 (100.0)	24,934 (62.6)
Age, *n* (%)		
≤75	38,534,953 (72.8)	12,247 (37.6)
>75	14,367,968 (27.2)	20,349 (62.4)
Sex, *n* (%)		
Female	29,928,520 (56.6)	18,982 (58.2)
Male	22,974,401 (43.4)	13,614 (41.8)
Race, *n* (%)		
Asian	844,228 (1.6)	262 (0.8)
Black	4,523,321 (8.6)	4520 (13.9)
Hispanic	958,465 (1.8)	828 (2.5)
White	45,495,610 (86.0)	26,512 (81.3)
Other	1,081,297 (2.0)	474 (1.5)
Urbanicity ^a^, *n* (%)		
Urban	39,656,002 (75.0)	22,313 (68.5)
Nonurban	11,897,208 (22.5)	9414 (28.9)
Income (Urban)		
Low	7,818,031 (14.8)	6904 (21.2)
Middle	15,468,091 (29.2)	8283 (25.4)
High	16,369,880 (30.9)	7126 (21.9)
With NO_2_^b^ Data, *n* (%)	48,224,895 (91.2)	25,694 (78.8)
With BRFSS ^c^ Data, *n* (%)	28,416,054 (53.7)	8106 (24.9)

Abbreviations: PM_2.5_ = particles with aerodynamic diameters <2.5 μm; NO_2_ = nitrogen dioxide. ^a^ Urbanicity data was available for 29,572 ZIP codes covering 97.5% of population. ^b^ NO_2_ data first became available in 2001. ^c^ BRFSS data first became available in 2002.

**Table 2:** Type 1 diabetes mortality hazard ratios (95% CI) associated with a 10 μg/m^3^ increase in 12- to 60-month moving average PM_2.5_, US 2000–2008.

Model ^b^	PM_2.5_
Exposure Window ^c^	
12-month	1.136 (0.993–1.301)
24-month	1.142 (0.971–1.343)
36-month	1.245 (1.043–1.486)
48-month	1.408 (1.160–1.710)
60-month	1.527 (1.235–1.889)

Abbreviations: CI = confidence interval; PM_2.5_ = particles with aerodynamic diameters < 2.5 μm. ^b^ Estimated using Cox PH models with strata for age (1-year age categories, with 90+ years old as one category), sex (male, female), race (White, non-White), and ZIP Code (39,804 ZIP codes), adjusted for ZIP code and state SES. ^c^ Subset of ZIP codes with complete data of 12- to 60-month moving average.

## Data Availability

The data that support the findings of this study are not publicly available due to confidentiality requirements set forth by the Centers for Medicare and Medicaid Services in our data use agreement. The original data used in this study can be requested independently from the Centers for Medicare and Medicaid Services (CMS.Gov).
